# Supplementary material for: Chromatin Accessibility Dynamics Reveal Conserved Transcriptional Regulatory Networks During Insect Metamorphosis in Harmonia axyridis and Drosophila melanogaster
Source: Biology (Basel). 2025 Jul 22;14(8):912. doi: 10.3390/biology14080912 (PMC12383559; doi:10.3390/biology14080912)
Supplement: Supplementary file 1 [file biology-14-00912-s001.zip › biology-3739016-Figure S1.pdf]

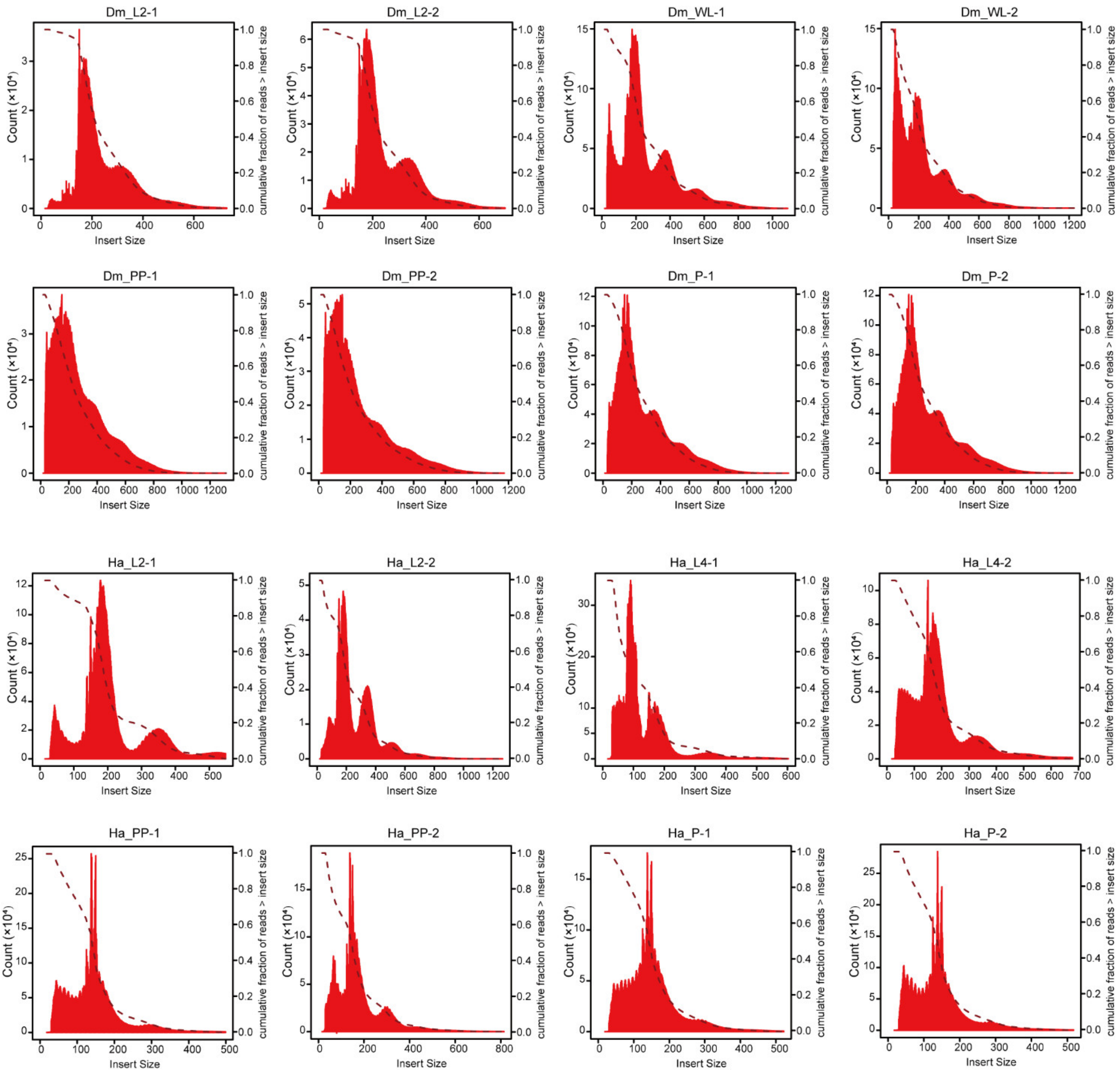

Figure S1. Insert size distribution of ATAC-seq fragments from *Drosophila melanogaster* (Dm) and *Harmonia axyridis* (Ha) samples. Each subplot represents an individual ATAC-seq library, with the x-axis indicating the fragment insert size (bp), the left y-axis showing fragment count (red histogram), and the right y-axis displaying the cumulative fraction of reads (black dashed line). Peaks around 50 bp correspond to nucleosome-free fragments, while subsequent peaks at ~200 bp and ~400 bp represent mono- and di-nucleosome-protected fragments, respectively. The observed fragment size patterns reflect the typical chromatin accessibility landscape, with sample-specific differences in nucleosome positioning and library complexity.
